# A Hypoxia-Related Long Non-Coding RNAs Signature Associated With Prognosis in Lower-Grade Glioma

**DOI:** 10.3389/fonc.2021.771512

**Published:** 2021-11-19

**Authors:** Qinglin Feng, Cheng Qian, Shibing Fan

**Affiliations:** ^1^ Department of Neurosurgery, Chongqing University Three Gorges Hospital & Chongqing Three Gorges Central Hospital, Chongqing, China; ^2^ Department of Cardiology, The Affiliated Hospital of Southwest Medical University, Luzhou, China; ^3^ Department of Neurosurgery & Chongqing Municipality Clinical Research Center for Geriatric Diseases, Chongqing University Three Gorges Hospital, and School of Medicine Chongqing University, Chongqing, China

**Keywords:** lower-grade glioma, hypoxia, long non-coding lncRNA, The Cancer Genome Atlas, prognosis

## Abstract

Accumulating evidence suggests that hypoxia microenvironment and long non-coding lncRNAs (lncRNAs) exert critical roles in tumor development. Herein, we aim to develop a hypoxia-related lncRNA (HRL) model to predict the survival outcomes of patient with lower-grade glioma (LGG). The RNA-sequencing data of 505 LGG samples were acquired from The Cancer Genome Atlas (TCGA). Using consensus clustering based on the expression of hypoxia-related mRNAs, these samples were divided into three subsets that exhibit distinct hypoxia content, clinicopathologic features, and survival status. The differentially expressed lncRNAs across the subgroups were documented as candidate HRLs. With LASSO regression analysis, eight informative lncRNAs were selected for constructing the prognostic HRL model. This signature had a good performance in predicting LGG patients’ overall survival in the TCGA cohort, and similar results could be achieved in two validation cohorts from the Chinese Glioma Genome Atlas. The HRL model also showed correlations with important clinicopathologic characteristics such as patients’ age, tumor grade, IDH mutation, 1p/19q codeletion, MGMT methylation, and tumor progression risk. Functional enrichment analysis indicated that the HLR signature was mainly involved in regulation of inflammatory response, complement, hypoxia, Kras signaling, and apical junction. More importantly, the signature was related to immune cell infiltration, estimated immune score, tumor mutation burden, neoantigen load, and expressions of immune checkpoints and immunosuppressive cytokines. Finally, a nomogram was developed by integrating the HRL signature and clinicopathologic features, with a concordance index of 0.852 to estimate the survival probability of LGG patients. In conclusion, our study established an effective HRL model for prognosis assessment of LGG patients, which may provide insights for future research and facilitate the designing of individualized treatment.

## Introduction

Lower-grade glioma (LGG), defined as World Health Organization (WHO) grade II/III gliomas, is a prevalent and aggressive type of primary intracranial tumors in adults (1). Despite the advances in neurosurgical resection and adjunctive therapy, a majority of LGG patients still undergo tumor recurrence and progression to glioblastoma (WHO grade IV), resulting in deteriorations in quality of life and survival outcomes (2, 3). This prognostic heterogeneity highlights the importance of molecular classification in the clinical management of LGG patients. Some molecular markers have been established for glioma subclassification, including the isocitrate dehydrogenase mutation (IDH) and the chromosomal 1p and 19q (1p/19q) co-deletion (4). In the era of precision medicine, however, these widely accepted factors are unlikely to provide sufficient insights for individual risk assessment of patients with LGG. Therefore, it is necessary to uncover novel biomarkers with excellent performance in predicting the prognosis and optimizing the treatment of LGG patients.

Hypoxia is a pivotal feature of malignant tumors that originated from the imbalance between accelerated tumor cell growth and insufficient intravascular oxygen supply (5). Mounting evidence has revealed the profound impacts of hypoxia on various tumor processes, including cell proliferation and differentiation, angiogenesis, invasion, metastasis, and immune infiltration (6–8). Hypoxia adaption, mainly mediated by the hypoxia-inducible factor (HIF) family, can promote tumor progression, treatment resistance, and poor prognosis in multiple malignancies (9). Recently, the implication of long non-coding RNAs (lncRNAs) in hypoxic signaling has become a new focus of attention in cancer research. LncRNAs belong to a subclass of RNA transcripts with longer than 200 nucleotides in length (10). Despite the lack of protein-coding potential, lncRNAs can regulate diverse of molecular and biological processes and contribute to tumorigenesis and tumor progression (11). To date, the role of lncRNAs in hypoxia-induced hallmarks has been explored in many cancer types including gliomas. For instance, lncRNA PDIA3P1 was reported to promote the hypoxia-induced mesenchymal transition by serving as a sponge of miR-124-3p in glioma (12). Also under hypoxic condition, LINC00475 silencing could inhibit the malignant behaviors of glioma through down-regulating AGAP2 (13). However, the prognostic utility of hypoxia-related lncRNAs (HRLs) has been not investigated in LGG patients.

In this work, we analyzed the transcriptome profiles from publically accessible databases and screened out HRLs using clustering and differentially expression analyses. Based on the gene expression data of candidate HRLs, a prognostic signature was established and then validated in patients with LGG. Furthermore, we also examined the associations of HRL signature with clinicopathologic features, biological functions, and immune microenvironment. Finally, a predictive nomogram was constructed to improve the clinical significance of the HRL model. The technology roadmap of our study was shown in **Supplementary Figure 1**.

## Materials and Methods

### Data Source and Processing

The RNA-sequencing transcriptome data and clinical characteristics of LGG cohorts were acquired from The Cancer Genome Atlas (TCGA, https://cancergenome.nih.gov) and the Chinese Glioma Genome Atlas (CGGA, http://www.cgga.org.cn) databases, including TCGA-LGG, CGGA-325, and CGGA-693. After removing tumor-adjacent samples and samples without complete survival information, a total of 913 patients with primary LGG were finally included in our study. The clinical data of these patients were summarized in **Supplementary Table 1**. For subsequent analysis, the transcriptome data were converted into transcripts per kilobase million (TPM) values with log2(x+1) transformation. Then, the gene identifications were annotated according to the GENCODE (https://www.gencodegenes.org, release 22) database and separated into mRNAs and lncRNAs. Those genes symbols with zero expression values in more than 5% of samples in each cohort were excluded from further analysis.

### Identification of Hypoxia Subtypes

Hypoxia-related mRNAs were collected from the gene sets “hypoxia;M10508” and “Cellular response to hypoxia;M26925” (14), which are available in the Molecular Signatures Database (MSigDB, https://www.gsea-msigdb.org/gsea/msigdb). To evaluate the hypoxia condition, we scored individual samples against the set of hypoxia-related mRNAs (termed “hypoxia enrichment score”) using single-sample gene set enrichment analysis (ssGSEA) with “GSVA” R package (15). Kaplan–Meier survival analyses were performed to confirm the impact of hypoxia status on LGG prognosis, using “survminer” R package. To identify different hypoxia subtypes in the TCGA-LGG cohort, consensus clustering according to the TPM values of hypoxia-related genes was performed with “ConsensusClusterPlus” R package (16). The optimum number of clusters was selected based on the consensus matrices and the cumulative distribution function (CDF) curves of consensus index. The clustering results were then evaluated using t-distributed stochastic neighbor embedding (t-SNE) algorithm (17). The survival outcomes, hypoxia enrichment score, and distribution of clinicopathologic features were compared between different clusters, including age, gender, tumor grade, IDH mutation, 1p/19q codeletion, and O6-methyl-guanine-DNA-methyltransferase (MGMT) methylation.

### Construction of HRL Prognostic Signature

The differentially expressed lncRNAs (DElncRNAs) between each two of the hypoxia clusters were detected using “limma” R package, with the cutoff criteria of |fold change|>2 and false discovery rate (FDR)<0.05. Candidate HRLs were defined as the common lncRNAs collected from Venn analysis of the differentially expression results. Then, LASSO penalized Cox regression model was developed to identify the core HRLs associated with patients’ survivals in the TCGA-LGG cohort using R “glmnet” package. This algorithm employed a penalty parameter λ.1se to prevent overfitting, which was generated from 10-fold cross validation (18). Finally, risk scores (namely “HRLscore”) were computed per patient by linear aggregation of the HRLs expression values weighted by the coefficients from LASSO algorithm.

### Evaluation of the Signature

To examine the prognostic accuracy of the HRLscore, we performed time-dependent receiver operating characteristic (ROC) analysis and calculated the area under the curve (AUC) using “survivalROC” R package (19). The AUCs of survival predictors were compared using “timeROC” R package (20). LGG patients were recruited into high- and low-HRLscore groups based on the optimal cut-off value of HRLscore derived from 5-year ROC curve. Kaplan-Meier survival analysis and log-rank test were conducted to explore the difference of survival between groups. Multivariate Cox regression analysis was applied to test the prognostic independence of HRLscore. We also performed stratified analyses to investigate the prognostic consistency of HRLscore across subpopulations. The HRLscore between patients with different clinicopathologic features were assessed.

### Gene Set Enrichment Analysis (GSEA)

GSEA (http://software.broadinstitute.org/gsea/index.jsp) (21) was performed to reveal the potential biological mechanisms associated with the HRLscore. In this study, we tested whether the hallmark gene sets (h.all.v7.4.symbol.gmt) were differentially expressed between the high- and low-HRLscore subgroups in the TCGA-LGG cohort. A hallmark gene set with nominal *p*<0.05 and FDR <0.25 after 1000 permutations was treated as statistically significant.

### Immune Microenvironment Analysis

The immune cell abundances of TCGA-LGG cohort were estimated using enrichment scores calculated by ssGSEA. The gene marker sets of 28 immune cells used for ssGSEA were downloaded from Charoentong’s study (22). We also applied the ESTIMATE algorithm (23) to quantify the immune score for each LGG sample. Tumor mutation burdens (TMBs) were calculated using “maftools” R package (24), and the neoantigen loads (NALs) were collected from a previous published study (25). In addition, the gene expression levels of immune checkpoints and immunosuppressive cytokines were investigated in different HRLscore groups.

### Development of Predictive Nomogram

The nomogram was generated to predict the 3- and 5-year survival rates by integrating the HRLscore and clinicopathologic characteristics *via* “rms” R package (26). We calculated the concordance index (C-index) to examine the predictive accuracy of the nomogram. Calibration curves was plotted to assess the concordance between predicted and actual survivals after bias control.

### Statistical Analysis

All statistical analyses were realized with R 3.6.0 software (The R Foundation for Statistical Computing, Vienna, Austria). Quantitative and qualitative data in two groups were compared using Wilcoxon rank-sum test and Chi-squared test, respectively. The association between two continuous variables was determined using Spearman correlation analysis. Student t-test was used to compare the C-index of the HRLscore and the multigene signatures derived from existing literatures. A *p* value of <0.05 was considered statistically significance.

## Results

### Hypoxia Subtypes in TCGA-LGG Cohort

**Figure 1:**
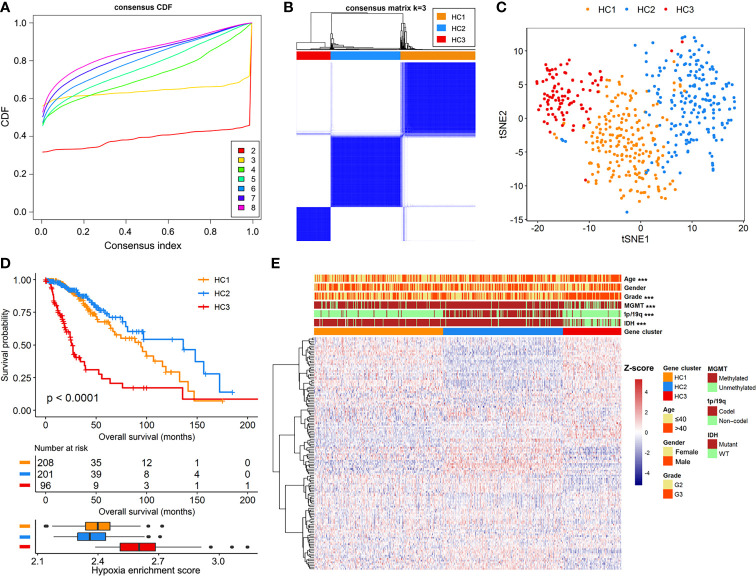
Identification of hypoxia-related subtypes in the TCGA-LGG cohort. **(A)** Cumulative distribution function (CDF) curves for k=2 to k=8. **(B)** Heatmap of consensus clustering matrix at k=3. **(C)** The classification into three subtypes validated by t-SNE analysis in the TCGA-LGG cohort. **(D)** Survival curves and hypoxia enrichment scores for the three hypoxia subtypes. **(E)** Heatmap of hypoxia-related mRNAs and clinicopathologic information across the three hypoxia subtypes.

We documented a total of 151 hypoxia-related mRNAs from the MSigDB, of which 142 were abundantly expressed in the LGG cohorts. The hypoxia enrichment score could recognize LGG patients with different overall survivals (**Supplementary Figure 2**), indicating the potential role of hypoxia in LGG development. Based on the expression profiles of hypoxia-related mRNAs, a consensus clustering algorithm was applied to mine different subtypes (cluster number k=2, 3, 4, 5, 6, 7, and 8) among the 505 LGG samples. At k=3, the CDF curve of consensus index score showed the flattest slope (**Figure 1A**), and the heatmap of consensus matrix had a relatively clear-cut boundary (**Figure 1B**). Thus, we recruited the LGG samples into three hypoxia-related clusters, namely HC1 (n=208), HC2 (n=201), and HC3 (n=96). The distribution patterns from t-SNE analysis were generally coordinated with the result of consensus clustering (**Figure 1C**), indicating that the three hypoxia subgroups were successfully separated from each other. Kaplan-Meier survival analysis revealed remarkable prognostic variations in the TCGA-LGG cohort (log-rank *p*<0.001, **Figure 1D**), with poorer overall survival for HC3 (median: 25.8 months) than HC1 (median: 94.5 months, log-rank *p*<0.001) and HC2 (median: 136.1 months, log-rank *p*<0.001). Accordingly, HC3 had the highest hypoxia enrichment score when compared with HC1 (*p*<0.001) and HC2 (*p*<0.001, **Figure 1D**), implying this cluster may be more hypoxic. In addition, there were significant differences of clinicopathologic features among the three clusters, including age, tumor grade, IDH mutation, chromosomal 1p/19q codeletion, and MGMT methylation (all *p*<0.001, **Figure 1E**). Taken together, based on the gene expression patterns, we detected three hypoxia-related subtypes that exhibited distinct survival outcomes and clinicopathologic characteristics in LGG patients.

### Construction of the HRL Prognostic Model

**Figure 2:**
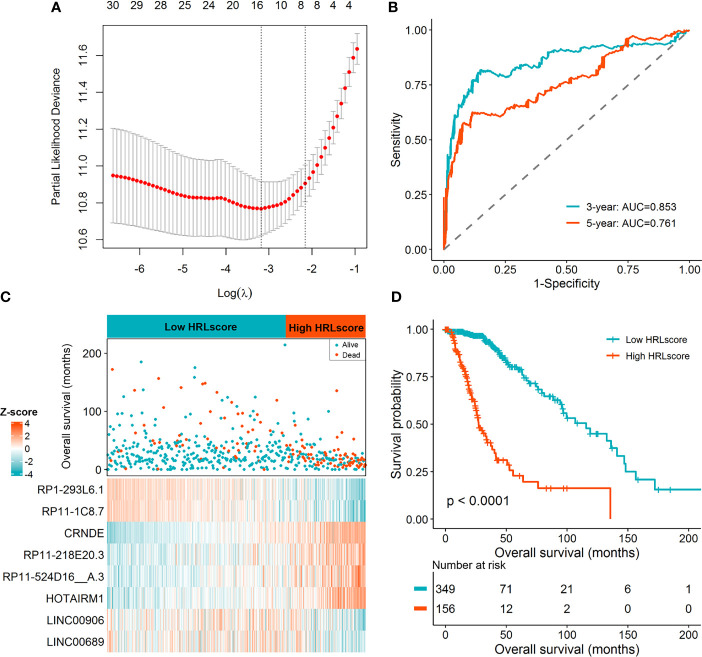
Derivation of the HRL signature in the TCGA-LGG cohort. **(A)** LASSO regression analysis with 10 cross-fold validation. **(B)** Time-dependent ROC curves for evaluating the prognostic performance. **(C)** Distribution of overall survival and expression of the eight informative HRLs between high- and low-HRLscore groups. **(D)** Survival curves for patients with different HRLscore.

Limma test was implemented to obtain the lncRNAs associated with the hypoxia patterns. Using the aforementioned significance threshold, we identified 364 DElncRNAs for the comparison of HC1 *versus* HC2, 777 DElncRNAs for the comparison of HC2 *versus* HC3, and 448 DElncRNAs for the comparison of HC1 *versus* HC3 (**Supplementary Figure 3**). Venn analysis of the differentially expression results resulted in 38 shared lncRNAs (**Supplementary Figure 3**), among which 31 were also profiled in the CGGA datasets. To prevent model overfitting, LASSO regression analysis was conducted for these genes and screened out eight HRLs as the key predictors of overall survival in the TCGA-LGG cohort (λ.1se=0.115, **Figure 2A**). The detailed information for the prognostic lncRNAs were listed in **Table 1**. The HRLscore for each patient was calculated as follows: HRLscore = (-0.0122 × expression of RP1-293L6.1) + (-0.0658 × expression of RP11-1C8.7) + (0.1509 × expression of CRNDE) + (0.0484 × expression of RP11-218E20.3) + (0.0714 × expression of RP11-524D16:A.3) + (0.0595 × expression of HOTAIRM1) + (-0.0610 × expression of LINC00906) + (-0.0181 × expression of LINC00689).

**Table 1:** The significant lncRNAs associated with overall survival in LASSO penalized regression.

Ensemble ID	Gene symbol	Genomic coordinate	HR	95% CI	LASSO Coefficient
ENSG00000234688	RP1-293L6.1	Chr 22: 36,703,918-36,721,472 (+)	0.799	0.753–0.848	-0.0122
ENSG00000271830	RP11-1C8.7	Chr 8: 103,481,266-103,481,619 (–)	0.776	0.729–0.826	-0.0658
ENSG00000245694	CRNDE	Chr 16: 54,918,863- 54,929,189 (–)	1.549	1.404–1.708	0.1509
ENSG00000258711	RP11-218E20.3	Chr 14: 50,956,259- 50,962,002 (–)	1.656	1.467–1.868	0.0484
ENSG00000261295	RP11-524D16:A.3	Chr X: 100,673,330-100,673,981 (+)	1.404	1.287–1.531	0.0714
ENSG00000233429	HOTAIRM1	Chr 7: 27,095,647- 27,100,265 (+)	1.390	1.287–1.502	0.0595
ENSG00000267339	LINC00906	Chr 19: 28,965,131- 28,970,874 (+)	0.820	0.756–0.890	-0.0610
ENSG00000231419	LINC00689	Chr 7: 159,006,522- 159,030,195 (+)	0.877	0.821–0.938	-0.0181

CI, confidence interval; HR, hazard ratio; LASSO, Least Absolute Shrinkage and Selection Operator.

Time-dependent ROC analyses were performed to determine the prognostic power of the HRLscore. In the TCGA-LGG cohort, the AUC of 3- and 5-year ROC curves were 0.853 and 0.761, respectively, demonstrating a good performance of the HRLscore in predicting the survival outcomes of LGG patients (**Figure 2B**). According to the best cut-off value of HRLscore derived from the 5-year ROC curve, we assigned the samples in the TCGA-LGG cohort into low-HRLscore (n=349) and high-HRLscore (n=156) groups. The survival status and lncRNAs expression profiles between groups were displayed in **Figure 2C**. Compared with patients with low HRLscore, those with high HRLscore had significantly higher hypoxia enrichment scores (median: 2.48 *versus* 2.37, *p*<0.001) and shorter overall survivals (median: 27.3 *versus* 115.7 months, log-rank *p*<0.001; **Figure 2D**).

### Validation of the HRL Prognostic Model

**Figure 3:**
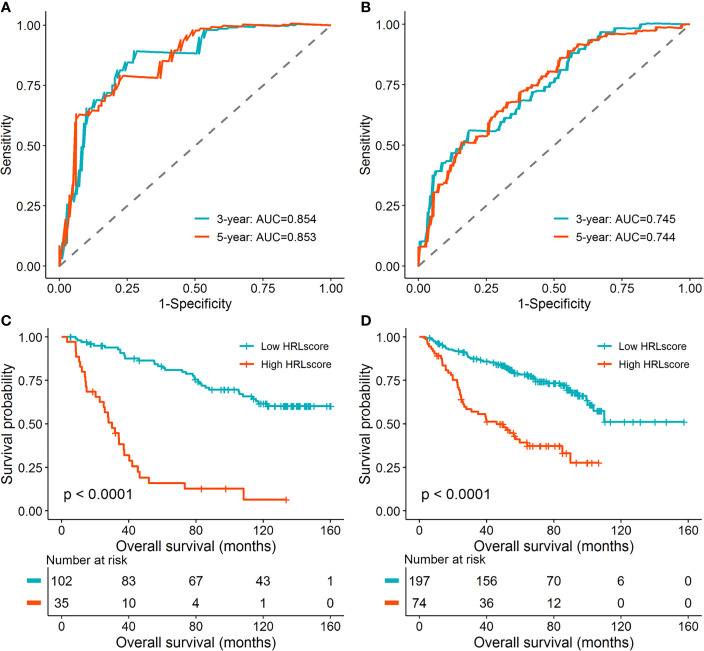
Validation of the HRL signature in the CGGA database. **(A)** Time-dependent ROC curves for patients in the CGGA-325 cohort. **(B)** Time-dependent ROC curves for patients in the CGGA-693 cohort. **(C)** Survival curves for patients in the CGGA-325 cohort. **(D)** Survival curves for patients in the CGGA-693 cohort.

To investigate the extrapolative accuracy of the HRL signature, we further verified it in the CGGA-325 and CGGA-693 cohorts. The HRLscore was produced *via* the same formula established in the TCGA-LGG cohort. Time-dependent ROC analysis indicated that the AUC of 3- and 5-year ROC curves were 0.854 and 0.853, respectively, for the CGGA-325 cohort (**Figure 3A**) and 0.745 and 0.744, respectively, for the CGGA-693 cohort (**Figure 3B**). Patients were then allocated into two groups using the optimal threshold of HRLscore from the 5-year ROC curve. The hypoxia enrichment scores were significantly higher in the high- *versus* low-HRLscore groups in both of the CGGA cohorts, indicating patients with high HRLscore may be more hypoxic. Similarly, compared with patients with low HRLscore, those with high HRLscore had obviously poorer overall survivals in both CGGA-325 (log-rank *p*<0.001; **Figure 3C**) and CGGA-693 (log-rank *p*<0.001; **Figure 3D**) cohorts. These findings demonstrated the prognostic robustness of the HRL model in patients with LGG.

### Clinical and Functional Implication of HRLscore

**Figure 4:**
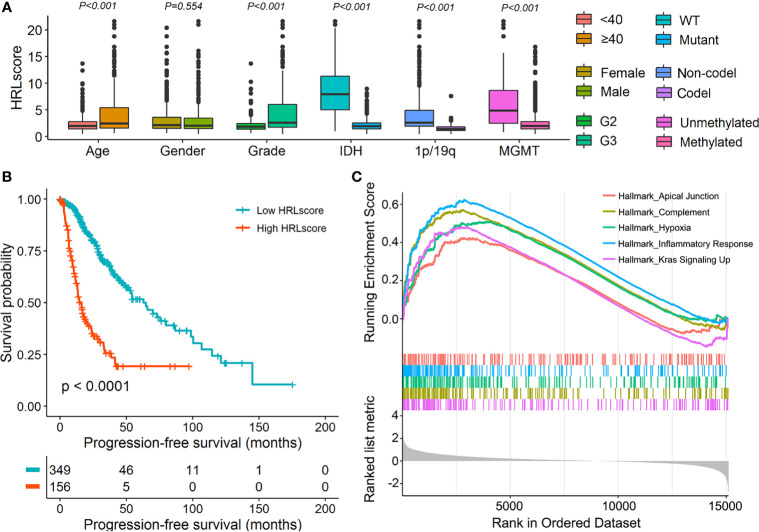
Clinical and functional implication of the HRL signature. **(A)** Comparison of the HRLscore between patients with different clinicopathologic features. **(B)** Progression-free survival curves for patients in the TCGA-LGG cohort. **(C)** Enrichment plots from GSEA for the comparison between patients with high and low HRLscore.

To assess the independence of HRLscore in survival prediction of LGG patients, we carried out multivariate Cox regression by introducing age, gender, tumor grade, IDH mutation, 1p/19q codeletion, MGMT methylation, and HRLscore as explanatory variables. It was found that the HRLscore was an independent predictor of overall survival in all of the three cohorts (HR=1.157, 3.379, and 4.017, respectively; **Table 2**). By performing stratified analyses in the TCGA-LGG cohort, we observed a consistent prognostic ability of HRLscore among patients with different clinicopathologic features (**Supplementary Figure 4**). To further clarify the clinical implication of the HRL signature, we tested the relationship between HRLscore and clinicopathologic variables in the TCGA-LGG cohort. The HRLscore was significantly different between subgroups of age (*p*<0.001), tumor grade (*p*<0.001), IDH mutation (*p*<0.001), 1p/19q codeletion (*p*<0.001), and MGMT methylation (*p*<0.001; **Figure 4A**). Moreover, the progression-free survivals were shorter in patients with high *versus* low HRLscore (median: 15.9 *versus* 62.9 months, log-rank *p*<0.001; **Figure 4B**). Finally, GSEA was employed to uncover the biological functions linked to the HRLscore. The results showed that this signature were mainly enriched in regulation of inflammatory response, complement, hypoxia, Kras signaling, and apical junction (**Figure 4C** **)**.

**Table 2:** Multivariate Cox regression analysis for overall survival in lower-grade glioma patients.

	TCGA (n = 505)			CGGA-325 (n = 137)			CGGA-693 (n = 271)		
Variables	HR	95% CI	*P*-value	HR	95% CI	*P*-value	HR	95% CI	*P*-value
Age*	1.048	1.031–1.066	<0.001	1.026	0.999–1.054	0.058	1.012	0.991–1.034	0.278
**Gender**									
Female	1.000 (reference)			1.000 (reference)			1.000 (reference)		
Male	1.327	0.912–1.931	0.140	0.757	0.439–1.303	0.315	2.188	1.214–3.946	0.009
**Grade**									
G2	1.000 (reference)			1.000 (reference)			1.000 (reference)		
G3	1.915	1.248–2.938	0.003	2.527	1.378–4.636	0.003	3.108	1.736–5.564	<0.001
**IDH**									
Wide-type	1.000 (reference)			1.000 (reference)			1.000 (reference)		
Mutant	0.681	0.349–1.329	0.260	1.511	0.707–3.230	0.287	0.589	0.274–1.266	0.175
**1p/19q**									
Non-codel	1.000 (reference)			1.000 (reference)			1.000 (reference)		
Codel	0.534	0.315–0.907	0.020	0.234	0.105–0.523	<0.001	0.425	0.176–1.025	0.057
**MGMT**									
Unmethylated	1.000 (reference)			1.000 (reference)			1.000 (reference)		
Methylated	1.085	0.643–1.831	0.759	0.857	0.482–1.526	0.601	0.613	0.363–1.034	0.067
HRLscore*	1.157	1.091–1.228	<0.001	3.379	1.576–7.243	0.002	4.017	1.705–9.460	0.001

CI, confidence interval; HR, hazard ratio; HRLscore, hypoxia-related lncRNA score.

*Analyzed as continuous variables.

### Prognostic Performance of HRLscore *Versus* Other Predictors

As shown above, some clinicopathologic features may also serve as predictors of LGG outcomes. Herein, we explored whether the HRLscore was superior to other parameters in terms of prognostic capacity. The AUC of HRLscore in survival prediction was generally higher than that of each clinicopathologic feature, and addition of the HRLscore to these variables could improve the prognostic performance (**Supplementary Table 2**). Furthermore, we compared the predictive accuracy of HRLscore *versus* several multigene signatures in existing publications by Zhang ret al. (27), Wang et al. (28), and Zhao et al. (29). The result demonstrated that the HRLscore was not inferior to other three prognostic models in the TCGA-LGG cohort (**Supplementary Figure 5**). In the CGGA cohorts, however, the HRLscore outperformed other signatures in predicting patients’ overall survival (**Supplementary Figure 5**).

### HRLscore and Immune Microenvironment

**Figure 5:**
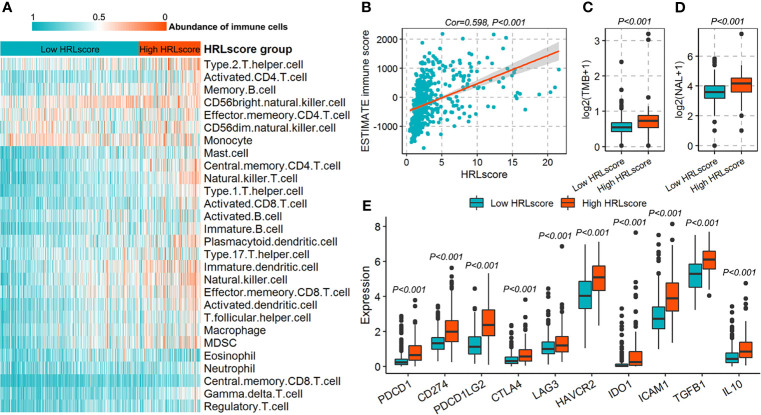
Immune characteristics for patients in the TCGA-LGG cohort. **(A)** Infiltration of 28 immune cell types between high- and low-HRLscore groups. **(B)** Correlation of HRLscore and ESTIMATE immune score. **(C)** Tumor mutation burdens (TMBs) between patients with high and low HRLscore. **(D)** Neoantigen loads (NALs) between patients with high and low HRLscore. **(E)** Expression of immune checkpoints and immunosuppressive cytokines between different HRLscore groups.

The fractions of 28 immune cell types in the TCGA-LGG cohort were estimated using ssGSEA algorithm. As a result, we observed that most of the immune cells infiltrated highly in the high-HRLscore group (**Figure 5A** and **Supplementary Figure 6**). There was a strong positive association between HRLscore and the immune score generated from ESTIMATE method (Spearman correlation coefficient=0.598, *p*<0.001; **Figure 5B**). We next evaluated the immunogenicity indices that were potentially linked to immunotherapy response. The high-HRLscore group exhibited increased TMBs (*p*<0.001; **Figure 5C**) and NALs (*p*<0.001; **Figure 5D**) when compared with the low-HRLscore group. Additionally, the expression levels of important immune checkpoints (*PDCD1*, *CD274*, *PDCD1LG2*, *CTLA4*, *LAG3*, *HAVCR2*, and *IDO1*) and immunosuppressive cytokines (*TGFB1* and *IL10*) were significantly higher in patients with high than with low HRLscore (all *p*<0.001; **Figure 5E**).

### Establishment of the Predictive Nomogram

**Figure 6:**
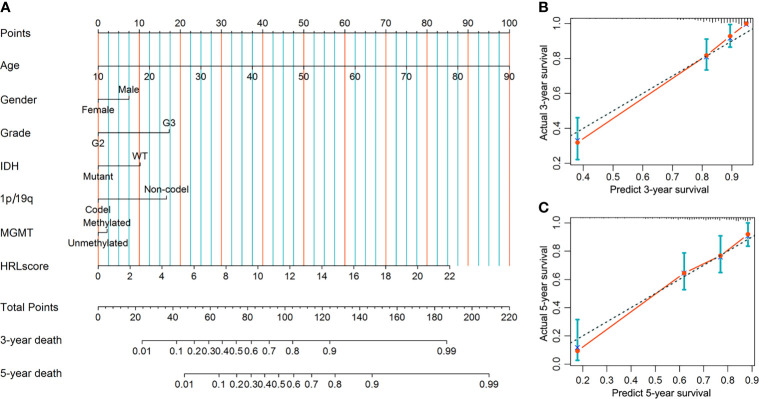
Construction and evaluation of the nomogram in the TCGA-LGG cohort. **(A)** Nomogram based on the HRLscore and clinicopathologic variables. **(B)** The 3-year calibration plot for the nomogram. **(C)** The 5-year calibration plot for the nomogram.

A predictive nomogram was developed to infer the 3- and 5-year likelihood of survival, which integrated our HRL signature and other clinical parameters. With this nomogram, each patient in the TCGA-LGG cohort was assigned a score and a predicted death rate (**Figure 6A**). Both the 3- and 5-year calibration charts showed a good concordance to the observed survival status (**Figures 6B, C**). Meanwhile, the C-index of the nomogram achieved 0.852 (95% confidence interval: 0.819–0.885), reflecting a high accuracy to predict the prognosis of LGG patients.

## Discussion

Hypoxia is an intrinsic hallmark of solid tumors and has been associated with tumor progression and poor prognosis in patients with glioma (30). In this study, the LGG samples could be clustered into three hypoxia subtypes according to the transcriptome profiles of hypoxia-related gene markers. These subtypes showed distinct hypoxia status, clinicopathologic features, and survival outcomes, indicating the importance of hypoxia in the molecular pathology of LGG. Since the hypoxia-lncRNA interactions have attracted much attention in cancer research (31), we further illustrated whether a HRLs-based model could effectively predict the prognosis of LGG patients. A total of eight lncRNAs (RP1-293L6.1, RP11-1C8.7, CRNDE, RP11-218E20.3, RP11-524D16:A.3, HOTAIRM1, LINC00906, and LINC00689) were selected to construct the HRL signature, which exhibited high accuracy and robustness in predicting patients’ survivals.

Although LGG is less aggressive than glioblastoma, increasing studies have demonstrated that it has a propensity towards progressing to higher grades, resulting in adverse outcomes (32). In our study, the HRLscore was higher in patients with advanced tumor grade and also served as a predictor of LGG progression in survival analysis. These findings reflect the potentially functional role of HRLs in the malignant transformation of LGG. In addition, we observed that unfavorable molecular features in glioma such as IDH wild-type, 1p/19q non-codeletion, and MGMT promoter unmethylation (33) were all associated with a higher HRLscore. Multivariate Cox regression suggested that the predictive value of HRLscore was independent of traditional clinical variables and molecular biomarkers. More importantly, the prognostic capacity of HRLscore was consistent across subpopulations with different clinicopathologic features. The above results indicated that our lncRNA signature is a reliable prognostic predictor for LGG patients. To further improve the clinical practicality of our signature, a nomogram was developed by integrating the HRLscore and clinicopathologic features, which had an excellent performance in the prognostic assessment of LGG.

The development of transcriptomics and bioinformatics has facilitated the discovery of novel biomarkers for cancer patients. Also for LGG, increasing studies have focused on establishing prognostic gene models based on mRNA, lncRNA, or proteomic approaches. For example, Zhao et al. (29) recently constructed a metabolism-related lncRNA-mRNA that could predict the clinical outcomes of LGG patients. In our study, we have compared the prognostic accuracy of HRLscore and other multigene models from previous publications (27–29). We found the HRLscore showed the highest C-index in CGGA cohorts, suggesting that our HRL signature may have a relatively high accuracy in the prognostic assessment of LGG. As lncRNAs function importantly in diverse of biological process (34), a multi-omic approach based on hypoxia hallmarks appears to have great prognostic potential for LGG patients.

Although thousands of lncRNA transcripts have been documented in genomic databases, the molecular functions for most of them remain under-investigated. To uncover the biological meanings behind our lncRNA signature, we performed GSEA between the different HRLscore groups. The result showed that in addition to hypoxia signaling, the HRLscore was also linked to inflammatory response, complement, Kras signaling, and apical junction. A growing body of studies suggests that inflammation is a critical contributor to the initiation and development of gliomas (35), and lncRNA CRNDE may trigger inflammation in glioma cell lines *via* toll-like receptor pathway (36). In gliomas, complement system has been shown to widely impact the malignant behaviors of tumor cells and regulate several microenvironmental components (37). Kras, a member of Ras oncogene family, is implicated in the pathogenesis of brain tumors such as glioblastoma (38) and pilocytic astrocytoma (39). Targeting Kras may inhibit the glioma cell proliferation and invasion *via* the downstream ERK signaling (40). In short, this evidence demonstrates that the HRL signature had significant influences on glioma development.

Since hypoxia is a major driver of tumor immune escape (41), we finally classified the immune microenvironment features associated with the HRLscore in LGG patients. Patients with high HRLscore tended to have more infiltrations of most immune cell types and increased ESTIMATE immune score, suggesting an immune heterogeneity within LGG tissues. Further analysis showed that the HRLscore was positively related to the gene levels of immune checkpoints and immunosuppressive cytokines. Based on these findings, we inferred that the immune activities in LGG microenvironment were suppressed by critical immune modulators (e.g., PD-1/PD-L1, CTLA-4, LAG3, TGF-β1, and IL10) (42–44), albeit with high invasion of immune cells. If indeed it is, LGG patients in the high-HRLscore group may benefit more from immunotherapy. Likewise, Chen et al. found that solid tumors with increased expression of PD-L1 and high infiltration of CD8+ T cells was more likely to benefit from blocking of immune checkpoints (45). Moreover, we observed that the high-HRLscore group exhibited increased TMBs and NALs, both of which have been proposed as predictors of immunotherapy response (46, 47).

Some limitations should not be ignored. First of all, this study is retrospective without details such as clinical therapy and surgery information, which may introduce some potential bias to our results. Secondly, although the HRL prognostic model was validated in CGGA database, its stability still needs more LGG samples for depth investigation. Thirdly, the molecular mechanisms associated with the eight HRLs in LGG should be uncovered in *in vivo* and *in vitro* experiments. Finally, the hypoxia-related mRNAs in MSigDB may not necessarily be accurate for LGG since they were derived from other tumor types.

Taken together, we developed and validated a HRL-based signature that could effectively predict the survival outcomes of patients with LGG. This prognostic model was correlated with important clinical pathologic features and showed a good capacity to characterize the immune microenvironment of LGG. These findings may provide useful targets for investigating the pathology and designing the individualized treatment of LGG.

## Data Availability Statement

Publicly available datasets were analyzed in this study. This data can be found here: TCGA database (https://cancergenome.nih.gov) and CGGA database (http://www.cgga.org.cn).

## Ethics Statement

Ethical review and approval was not required for the study on human participants in accordance with the local legislation and institutional requirements. Written informed consent for participation was not required for this study in accordance with the national legislation and the institutional requirements.

## Author Contributions

SF conceived and designed the study. QF and CQ collected and analyzed the data from public databases. QF drafted the paper, with key intellectual contents revised by SF and CQ. All authors contributed to the article and approved the submitted version.

## Funding

This study was supported by the grant from the Fundamental Research Funds for the Central Universities (Project No. 2021CDJYGRH-007).

## Conflict of Interest

The authors declare that the research was conducted in the absence of any commercial or financial relationships that could be construed as a potential conflict of interest.

## Publisher’s Note

All claims expressed in this article are solely those of the authors and do not necessarily represent those of their affiliated organizations, or those of the publisher, the editors and the reviewers. Any product that may be evaluated in this article, or claim that may be made by its manufacturer, is not guaranteed or endorsed by the publisher.
