# Supplementary material for: A Hypoxia-Related Long Non-Coding RNAs Signature Associated With Prognosis in Lower-Grade Glioma
Source: Front Oncol. 2021 Nov 19;11:771512. doi: 10.3389/fonc.2021.771512 (PMC8640178; doi:10.3389/fonc.2021.771512)
Supplement: Supplementary file 1 [file DataSheet_1.pdf]

Supplementary Materials

Supplementary Table 1. Clinical characteristics of patients with lower-grade glioma

Variables	TCGA (n=505)	CGGA-325 (n=137)	CGGA-693 (n=271)
**Age, n (%)**			
**≤40 yrs**	248 (49.1)	74 (54.0)	140 (51.9)
**>40 yrs**	257 (50.9)	63 (46.0)	130 (48.1)
**Gender, n (%)**			
**Female**	226 (44.8)	52 (38.0)	120 (44.3)
**Male**	279 (55.2)	85 (62.0)	151 (55.7)
**Grade, n (%)**			
**G2**	245 (48.5)	90 (65.7)	130 (48.0)
**G3**	260 (51.5)	47 (34.3)	141 (52.0)
**IDH, n (%)**			
**Wide-type**	94 (18.7)	35 (25.7)	65 (27.0)
**Mutant**	408 (81.3)	101 (74.3)	176 (73.0)
**1p/19q, n (%)**			
**Non-codel**	340 (67.3)	85 (63.0)	158 (66.1)
**Codel**	165 (32.7)	50 (37.0)	81 (33.9)
**MGMT, n (%)**			
**Unmethylated**	88 (17.4)	61 (48.8)	89 (41.2)
**Methylated**	417 (82.6)	64 (51.2)	127 (58.8)
**Survival status, n (%)**			
**Alive**	380 (75.2)	72 (52.6)	172 (63.5)
**Dead**	125 (24.8)	65 (47.4)	99 (36.5)

Supplementary Table 2. The AUC of HRLscore and clinical features in survival prediction for LGG patients

	TCGA		CGGA-325		CGGA-693	
**Variables**	**3-year AUC**	**5-year AUC**	**3-year AUC**	**5-year AUC**	**3-year AUC**	**5-year AUC**
**Age**	0.78 (0.71–0.85)	0.72 (0.63–0.81)	0.66 (0.53–0.79) *	0.65 (0.54–0.76) *	0.55 (0.46–0.65) *	0.53 (0.44–0.61) *
**Gender**	0.50 (0.43–0.57)*	0.54 (0.46–0.62)*	0.51 (0.41–0.61) *	0.51 (0.42–0.60) *	0.51 (0.44–0.58) *	0.52 (0.45–0.59) *
**Grade**	0.70 (0.64–0.76)*	0.70 (0.62–0.78)	0.79 (0.70–0.86)	0.76 (0.41–0.83) *	0.63 (0.57–0.70) *	0.62 (0.55–0.68) *
**IDH**	0.77 (0.71–0.83)	0.68 (0.63–0.74)*	0.74 (0.65–0.84) *	0.69 (0.61–0.78) *	0.72 (0.65–0.80)	0.65 (0.58–0.71) *
**1p/19q**	0.57 (0.51–0.63) *	0.61 (0.54–0.67) *	0.70 (0.63–0.77) *	0.74 (0.67–0.81) *	0.70 (0.65–0.74)	0.70 (0.65–0.76)
**MGMT**	0.64 (0.58–0.70) *	0.61 (0.55–0.67) *	0.54 (0.44–0.65) *	0.57 (0.47–0.67) *	0.55 (0.46–0.63) *	0.57 (0.49–0.65) *
**HRLscore**	0.85 (0.76–0.85)	0.76 (0.71–0.85)	0.85 (0.79–0.92)	0.85 (0.79–0.92)	0.74 (0.67–0.81)	0.74 (0.68–0.81)
**Clinical parameters**	0.80 (0.73–0.87)	0.75 (0.67–0.83)	0.68 (0.55–0.81)	0.67 (0.56–0.78)	0.59 (0.50–0.68)	0.54 (0.45–0.62)
**Clinical parameters+HRLscore**	0.84 (0.79–0.90)†	0.78 (0.70–0.85)†	0.70 (0.57–0.82)†	0.69 (0.55–0.82)†	0.61 (0.50–0.71)†	0.56 (0.46–0.65)†

* p<0.05 compared with HRLscore

† p<0.05 compared with the combination of all clinical parameters

Data were reported as AUC and its 95% confidence interval

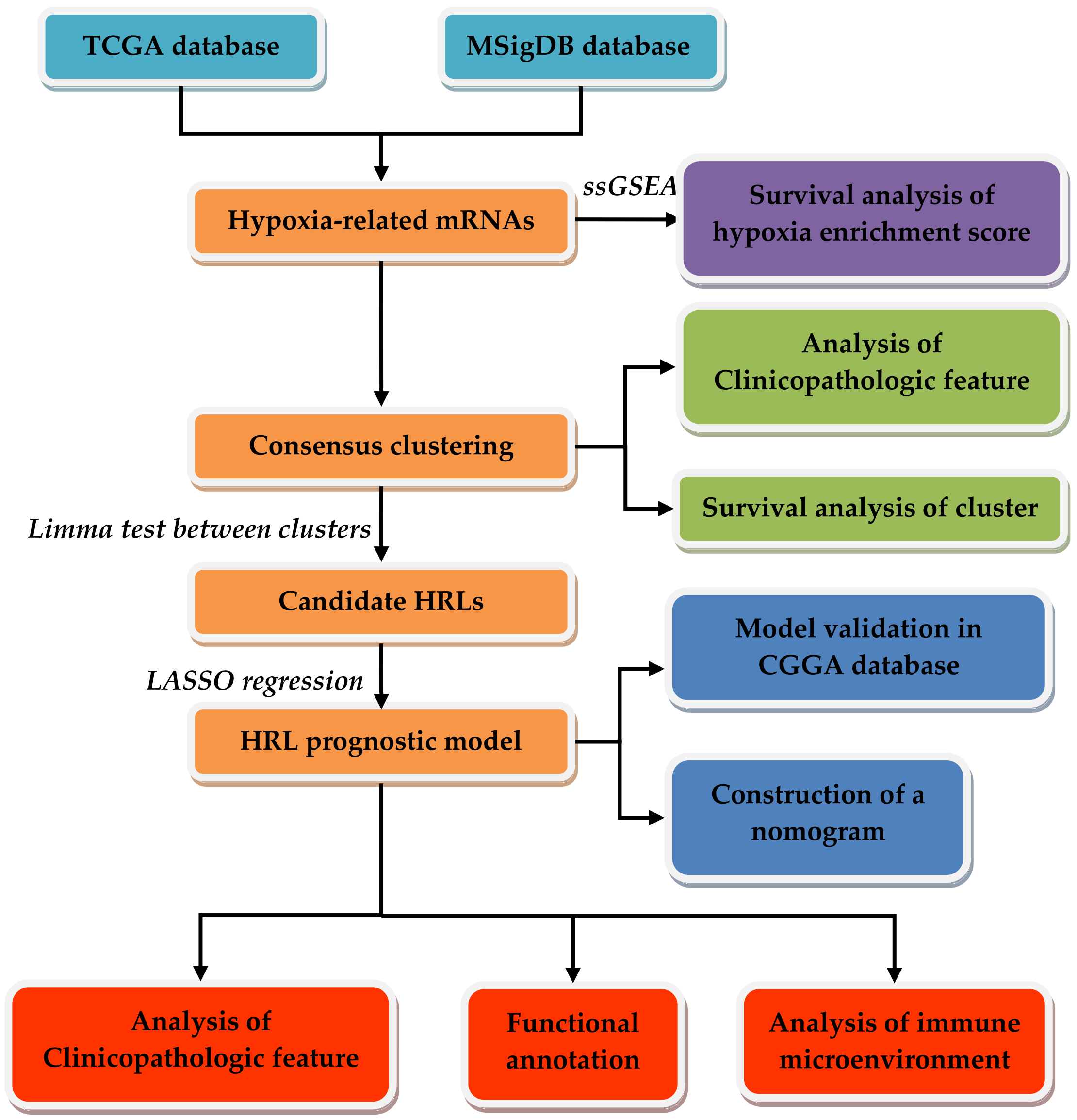

Supplementary Figure 1. The flow chart of data analyses.

Abbreviations: CGGA = Chinese Glioma Genome Atlas; HRLs = hypoxia-related long non-coding RNAs; TCGA = The Cancer Genome Atlas

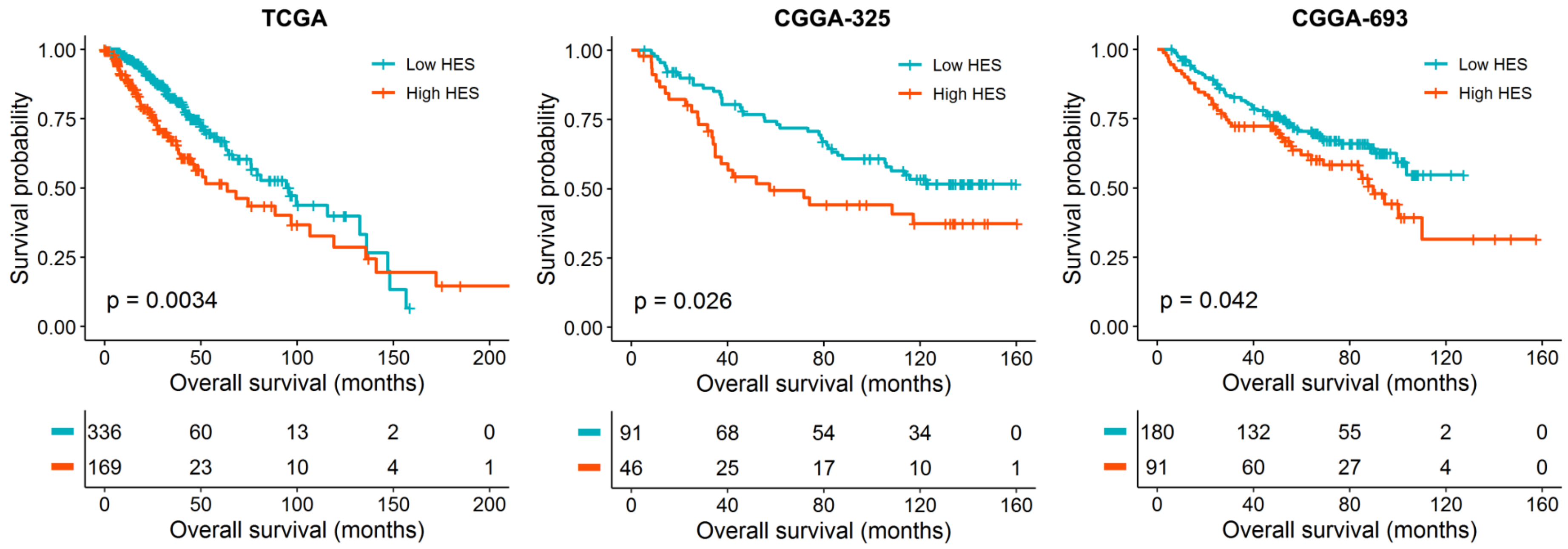

Supplementary Figure 2. The survival curves of LGG patients with different hypoxia enrichment score (HES). Patients in the upper tertile of HES were assigned into the high HES group, otherwise, the low HES group.

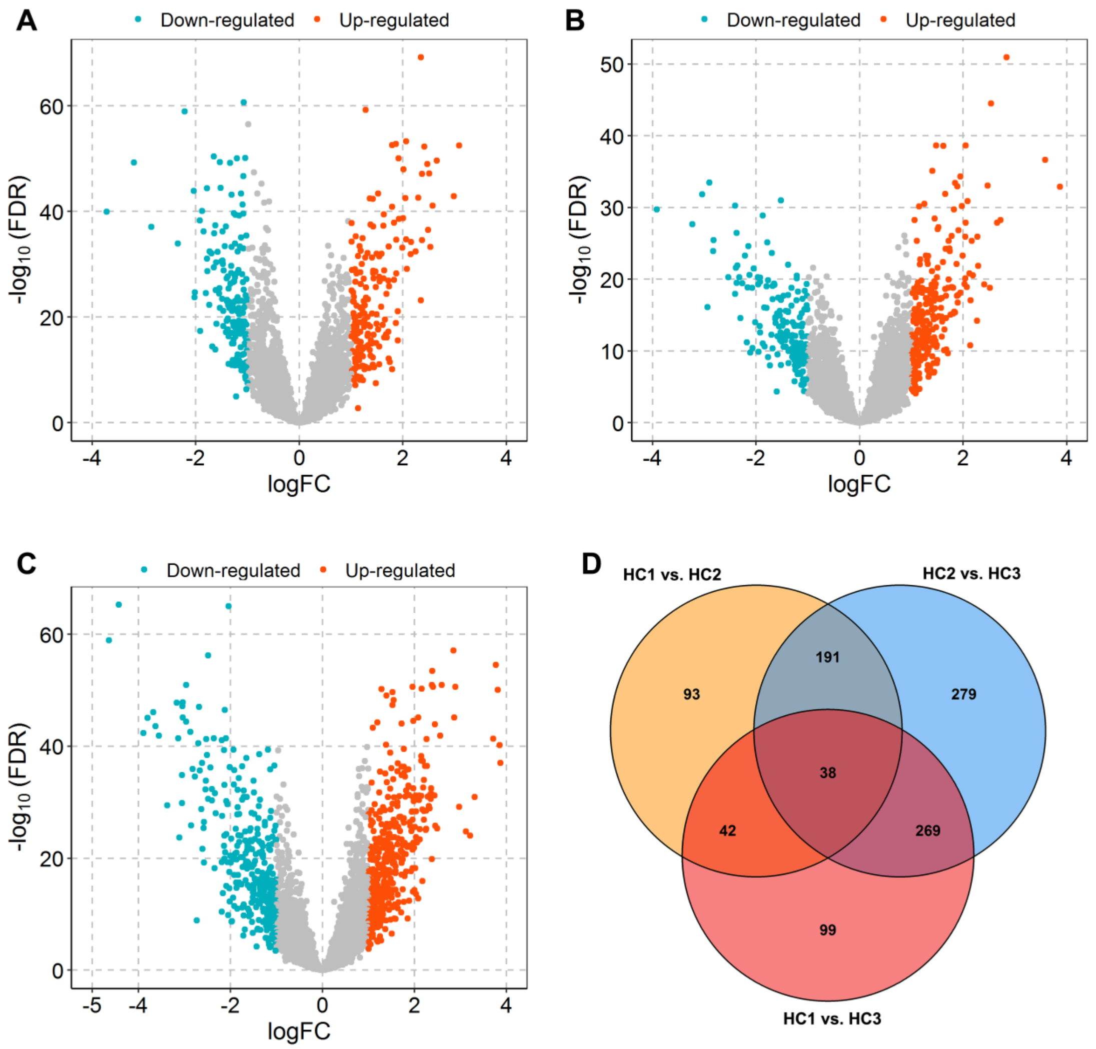

Supplementary Figure 3. Differentially expression analysis among hypoxia subtypes to identify hypoxia-related long non-coding RNAs (lncRNAs). A, B, and C, Volcano plots of differentially expressed lncRNAs in the comparison of HC1 versus HC2, HC2 versus HC3, and HC1 versus HC3, respectively. D, Venn diagram of the differentially expression results.

Abbreviations: FC = fold change; FDR = false discovery rate.

Variables	HR	95% CI
**Age**		
<40 yrs	1.421	1.264–1.596
≥40 yrs	1.224	1.173–1.278
**Grade**		
G2	1.461	1.291–1.653
G3	1.224	1.172–1.278
**IDH**		
Wide-type	1.136	1.070–1.206
Mutant	1.411	1.233–1.614
**1p/19q**		
Non-codel	1.264	1.212–1.318
Codel	1.917	1.296–2.835
**MGMT**		
Unmethylated	1.188	1.120–1.261
Methylated	1.348	1.267–1.433

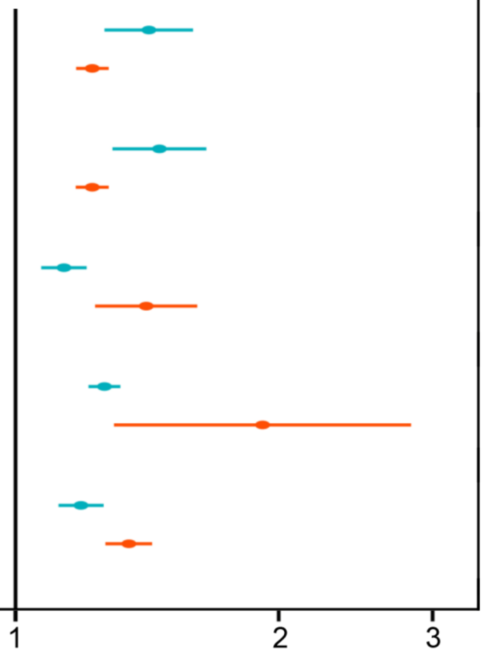

Supplementary Figure 4. The prognostic ability of HRLscore in patients with different clinicopathologic features.

Abbreviations: CI = confidence interval; HR = hazard ratio.

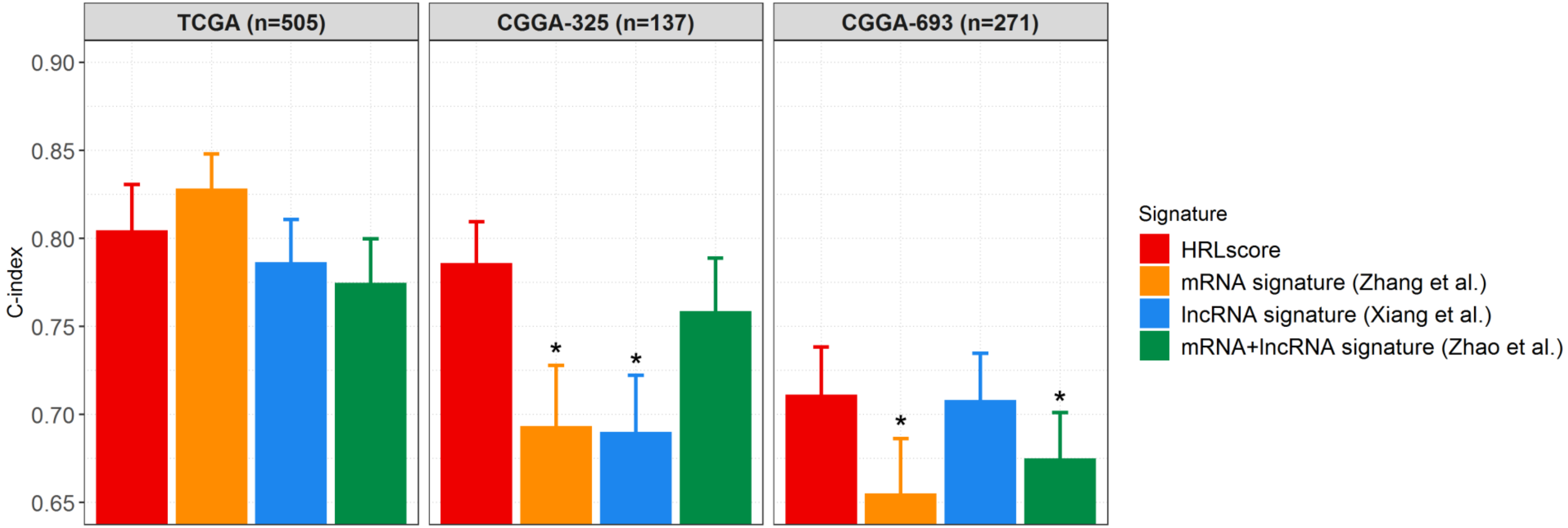

Supplementary Figure 5. The prognostic accuracy of HRLscore versus multigene signatures in some existing transcriptome studies

* p<0.05 compared with HRLscore

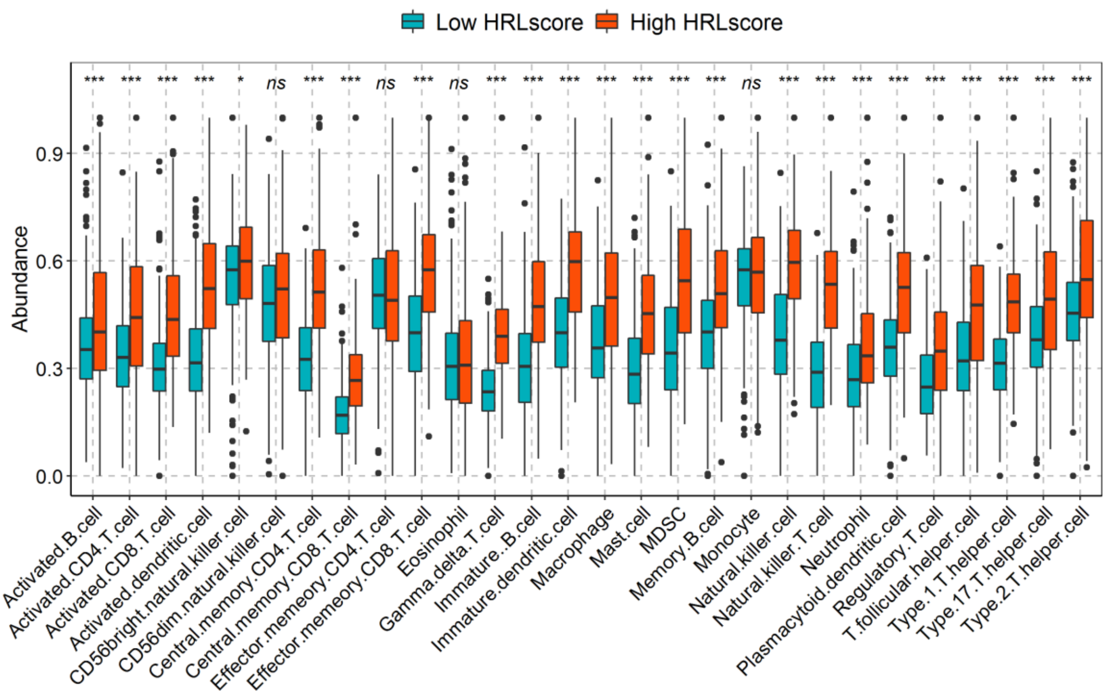

Supplementary Figure 6. The comparison of immune cell abundance between patients with high and low HRLscore.

Abbreviations: ns = no significance; * = p<0.05;*** = p<0.001.
